# Exploiting and Subverting Tor Signaling in the Pathogenesis of Fungi, Parasites, and Viruses

**DOI:** 10.1371/journal.ppat.1002269

**Published:** 2011-09-29

**Authors:** Cecelia A. Shertz, Maria E. Cardenas

**Affiliations:** Department of Molecular Genetics and Microbiology, Duke University Medical Center, Durham, North Carolina, United States of America; Duke University Medical Center, United States of America

## Tor Signaling Senses Nutrients and Growth Factors to Govern Pathways Involved in the Pathogenesis of Fungi, Parasites, and Viruses

In eukaryotes from yeast to humans, the Tor signaling cascade responds to nutrients and growth factors to orchestrate cell growth and proliferation. The central elements of this signaling cascade are the Tor protein kinases, which are the targets of the potent anti-proliferative and immunosuppressive natural product rapamycin [1]. Most organisms, including mammals, express a single Tor kinase; however, the yeasts *Saccharomyces cerevisiae* and *Schizosaccharomyces pombe* contain two Tor homologs [2], and three and four (two classical Tor kinases and two Tor-like kinases) have been identified in the protozoans *Leishmania major* and *Trypanosoma brucei*, respectively [3], [4]. The Tor kinases interact with other proteins to form two distinct and ubiquitously conserved complexes known as TORC1 and TORC2 (reviewed in [5]). Under optimal nutrient conditions, TORC2 drives actin polarization, whereas TORC1 promotes ribosome biogenesis and protein translation conducive to cell growth and proliferation while suppressing autophagy. In general, TORC1 is rapamycin sensitive in contrast to TORC2, which is resistant to rapamycin with the exception noted below for *T. brucei* [4], [6]. This report discusses recent studies uncovering emerging roles for Tor signaling in promoting fungal and protozoan pathogen growth and proliferation tailored to invade and colonize the host. Moreover, protozoans and viruses have also developed strategies to subvert the host Tor signaling cascade and thereby commandeer the translational machinery to evade the immune system and promote viral protein synthesis, respectively.

## Tor Regulates Cell–Cell Adhesion in *Candida albicans*

*Candida albicans* is the most common opportunistic fungal pathogen of humans, causing skin and mucosal infections as well as potentially fatal systemic infections. Cell–cell adhesion is necessary for *C. albicans* to form biofilms, an important feature of its pathogenicity repertoire. Interestingly, Tor1 regulates the expression of several cell wall– and hyphal-specific genes, including adhesins and their transcriptional regulators, which elicit biofilm formation in *C. albicans* [7].

Exposure of *C. albicans* to rapamycin results in upregulation of the transcriptional activators Bcr1 and Efg1 as well as downregulation of the transcriptional repressors Nrg1 and Tup1, which control adhesin genes [7]. These effects correlate with expression of hyphal-induced genes, including those encoding the adhesins Als1, Als3, and Hwp1 and the gene encoding the cell wall protein Ece1. Importantly, Als1, Als3, and Hwp1 mediate cellular adhesion to a variety of host surfaces and facilitate adhesion during biofilm formation (reviewed in [8]).

**Figure 1:**
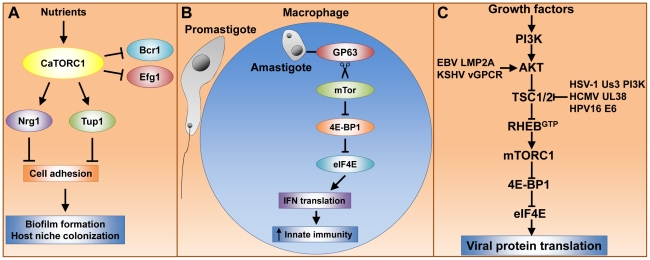
Tor signaling governs key processes that dramatically impact pathogenesis of fungi, parasites, and viruses. (A) caTor1 controls gene expression of adhesins and their upstream regulators, which elicit cell–cell adhesion, a process required for biofilm formation and host niche colonization. (B) The *L. major* GP63 cell surface protease promotes mTor cleavage and thereby mTORC1 inactivation. This event prevents mTORC1-dependent IFN type I translation, which dampens the host immune response and results in high parasite cell load. (C) Many human viral pathogens rely upon mTORC1 cap-dependent translation for replication. Growth factors activate mTORC1 via the phosphatidylinositol 3-kinase (PI3K), AKT, TSC1/2, Rheb signaling module (see text for details). Viruses have evolved protein factors capable of stabilizing the Rheb^GTP^ active form by either activating AKT or inactivating TSC2. Rheb^GTP^ activates mTORC1, enabling cap-dependent translation of early viral transcripts, which leads to viral replication.

Tor1 also regulates morphogenesis and cellular aggregation, which have implications for the pathogenicity and virulence of *C. albicans*. These effects appear to be at least in part mediated by Mds3, which is a regulator of morphogenetic processes such as the yeast to hyphal transition and biofilm formation [9], [10]. Although at present it is unclear how Mds3 acts, it has been suggested that Mds3 is a negative regulator of Tor1 [11]. Treatment with the Tor inhibitor rapamycin inhibits hyphal growth on solid media and causes extensive cellular aggregation and flocculation. These results are consistent with the model that Tor1 positively controls filamentation and negatively regulates cellular adhesion (Figure 1A) [7].

## Rapamycin Potently Inhibits Trypanosome Proliferation by Blocking TORC2 Assembly

*T. brucei* is a protozoan parasite responsible for causing ∼500,000 annual infections in Africa that result in sleeping sickness, with devastating socioeconomic effects. *T. brucei* has a complex life cycle that develops in two different hosts (the tsetse fly and vertebrates) and several niches within these hosts. Nutritional stress encountered in the insect triggers the development of the non-infectious procyclic trypomastigote into the infective metacyclic trypomastigote. In addition, nutritional stress also induces adhesion of the parasite to the digestive tract of the insect [12]. Once a trypanosome is delivered into the favorable conditions of vertebrate blood, the epimastigote develops into the bloodstream form of the parasite and rapidly proliferates to establish a successful infection (reviewed in [13]).

Recent studies have shown that Tor signaling profoundly impacts the growth and development of *T. brucei*. Two classical Tor1 and Tor2 proteins, as well as two Tor-like proteins, have been identified in *T. brucei* [4]. The tbTor1 kinase associates with TORC1 and controls protein synthesis and cell size while the Tor-like 1 protein regulates autophagy [4], [14], [15]. The tbTor2 kinase populates TORC2 and, in contrast to the paradigm established in other organisms, tbTORC2 is sensitive to rapamycin, whereas tbTORC1 is resistant to rapamycin. tbTORC2 is involved in driving polarized cell growth, endocytosis, and cytokinesis [4].

Trypanosome cells have a highly polarized microtubular network that enables endocytosis and exocytosis to occur at a single site called the flagellar pocket; any perturbation of this network compromises endocytosis with fatal consequences for the organism. Accordingly, either tbTor2 depletion or rapamycin exposure (which prevents tbTORC2 formation) results in aberrant cell morphology with an enlarged flagellar pocket, actin cytoskeleton depolarization, impaired endocytosis, and a block to cytokinesis [4]. Interestingly, rapamycin treatment of the *T. brucei* bloodstream form cultured in vitro markedly inhibits cell proliferation. Thus, it has been suggested that therapy with less immunosuppressive rapamycin analogs could be highly efficacious against trypanosomiasis [4].

## *Leishmania major* Hijacks the Host Translational Machinery via mTor Proteolysis

The protozoan parasite *L. major* is the etiologic agent for leishmaniasis and, similar to trypanosomes, develops as promastigotes in the midgut of sandflies and as amastigotes within macrophages of the vertebrate host. *L. major* contains three Tor homologs, at least one of which (Tor3) is required for macrophage infectivity and virulence [3].

mTOR-dependent translation of type I interferon (IFN) is critical for triggering host innate immune responses to defend against infection, including those caused by parasites [16], [17]. Interestingly, *L. major* hijacks the host translational machinery via disruption of mTor signaling, thereby enhancing parasite infectivity [18]. The *L. major* surface glycoprotein GP63, which exhibits Zn-dependent protease activity, is associated with *L. major* virulence. GP63 directly or indirectly promotes mTor cleavage, resulting in mTORC1 inhibition. This event prevents mTORC1-dependent phosphorylation and inactivation of host 4E-binding protein 1 (4E-BP1). Activated 4E-BP1 forms a physical complex with the mRNA cap binding factor eIF4E (elongation initiation factor 4E), thereby blocking the ability of eIF4E to assemble into the eIF4F complex and cap-dependent translation (reviewed in [19]). In parallel, type I IFN expression, which is deployed by the host immune system to defend against *L. major*, is decreased, though it is not yet clear what directly causes this phenomenon. Moreover, either rapamycin treatment or 4E-BP1 and 4E-BP2 knockout results in increased type I IFN expression and reduced *L. major* parasite load in mice. Thus, host mTORC1 is inactivated by *L. major* GP63 to promote parasite survival within host macrophages (Figure 1B). This model has led to the suggestion that host 4E-BP1/2 could be targeted therapeutically to treat leishmaniasis [18].

## Viruses Activate mTor-Dependent Translation to Ensure Infection

Viruses depend on the cellular protein synthesis machinery for viral protein translation. In particular, poxivirus, adenovirus, and human herpes virus mRNA translation is heavily dependent upon mTor to inactivate the repressor 4E-BP1 and thus enable eIF4F cap-dependent translation (reviewed in [19], [20]). Growth factors activate mTor via the AKT-kinase, TSC1/2, Rheb-GTPase signaling module (Figure 1C). In response to growth factors, AKT phosphorylates the tuberous sclerosis heterodimer TSC1/2, thereby inactivating its GTPase activating protein (GAP) activity, which results in the active Rheb^GTP^ form and, in turn, mTor activation (reviewed in [21]). Interestingly, herpes and other viruses have developed multiple mechanisms to activate mTor and ensure sustained viral protein translation (Figure 1C). Epstein-Barr virus (EBV) and Kaposi's sarcoma herpes virus (KSHV) encode the proteins LMP2A and G protein–coupled receptor vGPCR, respectively, which activate AKT [22], [23]. The human cytomegalovirus (HCMV) UL38 protein and the human papilloma virus (HPV) E6 oncoprotein antagonize TSC2 [24].

Remarkably, the herpes simplex virus (HSV-1) Us3 kinase acts as an AKT surrogate capable of phosphorylating and inactivating TSC2, resulting in constitutive mTORC1 activation [25]. These results suggest that mTor inhibition should prevent viral replication and, in fact, the mTor kinase catalytic site inhibitor Torin1 potently blocks herpes virus replication, particularly during the early phase of viral infection [26], [27]. The Torin1 studies also revealed that the role of mTor activation during HCMV infection is not confined to inactivating 4E-BP1 or exclusive to the rapamycin-sensitive mTORC1, and additional targets within the mTORC1 and mTORC2 pathway components await elucidation.

## Future Directions

The dramatic impact of nutrients as the triggers for morphogenic transitions that promote infection and host colonization in fungi and parasites, as well as the dependence of viral replication on the cellular translational machinery, prompted studies to investigate the roles of the Tor signaling cascade in these diverse infectious processes. Not surprisingly, the exciting results with pathogenic fungi, parasites, and viruses have thus far opened new avenues for further study. Recent studies with the *Salmonella* effector protein AvraA, which is conserved in *Yersinia* pathogenic species, suggest that bacteria have also developed strategies to target host signaling cascades (including mTor) to control gene expression and promote virulence [9]. Moreover, there is growing evidence that Tor-regulated processes such as autophagy have dramatic impact on virulence. In particular, it is well established that autophagy can destroy invading pathogens (a process also known as xenophagy) and enhances innate immunity, protecting cells and organisms against infection by a wide range of pathogens, including bacteria, viruses, and parasites (reviewed in [28]). Thus, we can expect that future studies in these arenas will continue to illuminate novel aspects of Tor signaling in microbial pathogenesis that will stimulate the development of new Tor-based therapies to combat infection.
